# 

**Published:** 1888-06-09

**Authors:** 


### Special Notice to Newsagents, Advertisers, and the Trade.

### CHANGE OF OFFICE AND PUBLISHER.

Notice is hereby given that the Office of The Hospital will henceforth be at 38, Old Jewry (Cheapside end), E.C., to which address all communications should be sent. Mr. A. Doig has been appointed Advertising Agent, and Mr. J. H. S. Hanning, Manager. All Cheques and Post Office Orders should be made payable to J. H. S. Hanning, 38, Old Jewry, E.C. crossed Lloyds, Barnetts, and Bosanquets Bank (Limited).

### To Secretaries and Matrons.

The Editor will be glad to have a copy of the Regulations, and also of every Report issued from the Press of Asylums, Hospitals, Convalescent and Nursing Institutions, as well as those of other Charities, so soon as they are published, and an early copy in duplicate of all appeals for funds. Notices of Annual and other Meetings, Festival Dinners, etc., will be welcomed. All such communications should be addressed to *The Editor, The Lodge, Porchester Square, London, W.* Any superintendent, secretary, matron, or other chief official who will regularly send such documents and information will be registered to receive free of cost a cover for binding The Hospital in half-yearly volumes on sending name and address to the publisher.

### AMUSEMENTS WITH PROFIT FOR ALL AGES.

**Rules.**—All answers must be addressed to the PRIZE EDITOR, THE HOSPITAL, 38, Old Jewry, Cheapside, London, E.C., not later than the *first* post on Thursday next. The full name and address must in all cases be given, but a *nom de plume* may be added for publication. Only one side o' the paper must be written on.

### FOR MEN AND WOMEN.

**Rule.**—Competitors can enter for *all* quarterly competitions, but no competitor may take more than *two quarterly prizes* during the year.

### First Quarterly Poetical Competition.

Commenced May 26th, 1888. Ends August 25th, 1888.

This quarter a PRIZE of ONE GUINEA will be given to the competitor who gains the highest number of marks for original poems, on any subjects suitable for insertion in this paper. All contributions sent in will be credited according to merit eighteen marks being the highest score for any one contribution. This competition to alternate weekly with the set acrostic competition.

### Acrostic Competition.

This quarter the original acrostic competition will be discontinued, and A PRIZE of HALF-A-GUINEA will be given to the competitor who gains the highest score for solution of acrostics set. *One mark* only will be given to the competitors who solve all the lights of each acrostic.

Exception will be made in the case of *quotation acrostics*, when *two marks* will be given, *one* for the correct solution of all *lights*, and *one* mark for the *source* of the quotations.

This week credit will be given for

No. 1. D UBLE ACROSTIC

Upon a lovely island which the sea
Embraced with his green arms most lovingly
My primals dwelt within her palace cave,
Whose rough-hewn pillars bore an architrave
Of weathered rock thus Nature loves to build,
And troubleth not herself to paint or gild.
Sweet and lovely as the flowers in May,
My primals watched the sea from day to day,
While once she looked, fast to her eager gaze,
A boat came on the sea, shrouded in haze.
As it approached she raised her charming head,
And to my finals in the boat she said
"Much thou hast wandered, much hast been oppresṭ,
Me it is thine to love, and here to rest;
Come to my fair isle, and there abide,
Shunning the treacherous and destroying tide."
And thus she chanted all along the shore,
As the swift bark to land my finals bore.

1. A ancient island, you now see its modern name,
By Corinth founded, it obtained wide-reaching fame,
Through her disputes, a war for many years did flame.

2. Though the dose be small, the poison is great,
Potent your thirst and most insatiate.

3. Many and fierce have been the battles fought for me;
By all republics loved; a foe to tyranny.

4 With these the savage tribes rush on against their foe,
With these they cheer each on as to the fight they go.

5 I am French and Latin and can never decrease,
As I'm a positive sign and always increase.

6. Without a grain of this, all labour is as nought,

It ought to be innate, 'tis not easily taught.

7. It touched the heavens with its top, the ancients say,
And here a mighty deity held regal sway.

### Answers.

No. 7. DOUBLE ACROSTIC.

| | | | |
|---|---|---|---|
| W | itlaf | F. | Longfellow. |
| S | pide | R. | "Winter's Tale," ii. 1. |
| H | ypochondri | A. | Thomson "Castle of Indolence." |
| A | ubur | N. | Goldsmith. |
| K | leptomania | C. | Parnell, "The Hermit." |
| E | nnu | I. | Shelley. |
| S | cale | S. | Longfellow, "Poet's Calendar." |
| P | et Lam | B. | Wordsworth. |
| E | rek | A. | Byron, "Childe Harold," iv. 81. |
| A | sceti | C. | Byron |
| R | odrig | O. | Lockhart, "Spanish Ballads." |
| E | to | N. | Gray "Ode on a Distant Prospect," etc. |

Correct answers have been received from Dunce.

### Results of Second Quarterly Original Acrostic Competition.

*Brownie* having gained the highest score for original acro tics during the past quarter, we award the prize of one guinea to Rev. J. E. L. Nowers, 3, Comeragh-road, West Kensington.

### Results of Second Quarterly Set Acrostic Competition.

*Dunce* is the winner of the Second Quarterly Set Acrostic Competition we therefore forward the prize of 10s. 6d. to Mrs. Young, 31, Perham-road, West Kensington.

### THE WORD COMPETITION.

Third Quarterly Competition commenced May 5th, 1888; ends August 4th, 1888.

Three prizes of one guinea, 15*s*. and 10*s*. 6*d*. will be given each quarter to the three competitors who obtain the highest number of marks by making the largest number of words derived from the word or phrase set for anatomical dissection; that for this, the *fifth* week of the quarter, being "COERCION."

OBSERVE.—Proper names, abbreviations, foreign words, words of less than four letters, and repetitions are barred; plurals, and past and present participles of verbs are allowed. Nuttall's *Standard* dictionary only to be used.

N.B.—All words sent in must be arranged *alphabetically*, with correct total affixed.

### Results of Third Week.

*Cricket.*—Lauriston (20), Tinie (19), Patience (19), Lightowlers (19), Dollie (18), Carmen (17), Brisbane (17), Dodo (17), Sym (16), Reldas (15), Puff (15), Geranium (13), Ione (12).

### THE CHILDREN'S CORNER.

PRIZES.
FOR MOST CORRECT ANSWERS TO PUZZLES.
This Commenced October 1st, 1887.
One Pound a Month.
A PRIZE OF THREE GUINEAS

to the Competitor who shall have sent in the largest number of correct or best answers during the twelve months.

### Puzzles—First Week.

No. 1. GEOGRAPHICAL CHARADE.—My *first* is two numerals; my *second* the nineteenth letter of the alphabet; my *third* a vowel; and my *whole* an American State.

No. 2. TRANSPOSITION.—

If part of a foot with care you transpose,
The answer you'll find just under your nose.

No. 3 DIAMOND PUZZLE.—1. A consonant. 2. A metal. 3. Places where it is found 4. A heathen goddess. 5. A bird. 6. A clinging plant. 7. A vowel.

a a e e e i i i i m m m n n n n o r r s v v h t y

No. 4. ENIGMA.—A bird and what a horse does not like, will give the name of a flower.

No. 5. CONUNDRUM.—When was there the same difference between Algiers and Malta as between light and darkness.

### Results of Third Week Puzzles.

No. 9. CHARADE.—Sal-mon = Salmon.

No. 10. SQUARE WORD.

| | | | |
|---|---|---|---|
| C | R | O | W |
| R | O | S | E |
| O | S | S | A |
| W | E | A | L |

No. 11. DOUBLE ACROSTIC.

| | | |
|---|---|---|
| L | air | S |
| A | stonishmen | T |
| D | ian | A |
| D | onizett | I |
| E | a | R |
| R | uben | S |

No. 12. ENIGMA —A pair of spurs.

*All right.*—Nunkey, A. C. K. *Three right.*—Jumbo, Pickles, Dame Durden Scrooge, Geranium, Gem. *Two right.*—Plummy Fluff, Spider. *One right.*—Happy Eliza, Judy.

### Conditions.

I. Every paper sent in must be clearly written, and one side only must be used. All competitors must mark at the head of their papers the number of their competition.

II. Each paper must be signed by the author with his or her real name and address. A *nom de plume* may be added, if the writer does not desire to be referred to by us by his real name. In the case of all prizewinners, however, the real name and address will be published.

III. All communications relating to prize competitions should be addressed to "The Prize Editor, THE HOSPITAL, 38, Old Jewry, London, E.C." Competitors are requested not to include in letters, etc., to the Prize Editor communications on matters of other business. All letters must be fully prepaid.

IV. The Prize Editor of THE HOSPITAL is the sole judge of the competitions, and his decision is in all cases final and without appeal.

V. The Prize Editor reserves the right to publish any paper sent in, whether it receives a prize or not. He does not hold himself responsible for any MSS. sent, nor can he undertake to return rejected competitions.

VI.—All children resident at school are requested to forward their *home* address in addition to the school one, when entering for the "Children's Corner" Competitions.

**Notice to all Correspondents**—N.B—All letters referring to this page, which do not arrive at 38, Old Jewry, Cheapside, London, E.C., by the *first post on Thursdays*, and are not addressed PRIZE EDITOR, will in future be disqualified and disregarded.

No one will be permitted to win the Monthly Prizes twice in any one year, the Annual Prize being offered especially to prevent this.

